# Trends in Prevalence, Awareness, Treatment, and Control of Diabetes Mellitus in Mainland China from 1979 to 2012

**DOI:** 10.1155/2013/753150

**Published:** 2013-10-28

**Authors:** Min-zhi Li, Li Su, Bao-yun Liang, Jin-jing Tan, Qing Chen, Jian-xiong Long, Juan-juan Xie, Guang-liang Wu, Yan Yan, Xiao-jing Guo, Lian Gu

**Affiliations:** ^1^First Affiliated Hospital, Guangxi University of Chinese Medicine, Nanning, Guangxi 530023, China; ^2^School of Public Health of Guangxi Medical University, Nanning, Guangxi 530021, China; ^3^Department of Internal Neurology, First Affiliated Hospital, Guangxi University of Chinese Medicine, 89-9 Dongge Road, Nanning, Guangxi, 530023, China

## Abstract

Diabetes mellitus (DM) is one of the primary causes of premature death and disability worldwide. We performed a systematic review and meta-analysis of the published literature regarding the trends in prevalence, awareness, treatment, and control of diabetes mellitus in mainland China. PUBMED, EMBASE, Chinese Biomedical Database, China National Infrastructure database, Chinese Wan Fang database, and Chongqing VIP database were searched. Fifty-six eligible studies were included. Increasing trends in the prevalence, treatment, and control of diabetes in mainland China from 1979 to 2012 were observed. The pooled prevalence, awareness, treatment, and control of diabetes mellitus were 6.41%, 45.81%, 42.54%, and 20.87%, respectively. A higher prevalence of diabetes mellitus was found in urban (7.48%, 95%CI = 5.45*~*9.50) than rural (6.53%, 95%CI = 4.30*~*8.76) areas. Furthermore, an increasing chronological tendency was shown in different subgroups of age with regard to the prevalence of diabetes. A higher awareness of DM was found in urban (44.25%, 95%CI = 32.60*~*55.90) than rural (34.27%, 95%CI = 21.00*~*47.54) populations, and no significant differences were found in the treatment, and control of diabetes among the subgroups stratified by gender and location. From 1979 to 2012, the prevalence, treatment, and control of diabetes mellitus increased; nevertheless, there was no obvious improvement in the awareness of diabetes.

## 1. Introduction

 With the rapid economic development, elevated standard of living, dietary shifts, lifestyle alterations, and aging, diabetes mellitus (DM) has become an important public health problem worldwide [1–3], which is estimated to be the third most challenging disease threatening public health after malignant tumors and cardiocerebral vascular diseases [4]. It has been estimated that the global number of individuals with diabetes will double from 171 million in 2000 to 366 million in 2030 among adults aged ≥20 years [5]. Data from European countries have indicated that the health care expenditure for patients with diabetes mellitus was significantly higher than for those who were not diagnosed with this disease [6–8]. Also, in the United States, it has been estimated that approximately 17.5 million people were diagnosed with insulin-dependent diabetes mellitus (IDDM) or non-insulin-dependent diabetes mellitus (NIDDM) in 2007, and the total annual cost in higher medical costs and lost productivity was estimated to be $174.4 billion [9], including $159.5 billion for the 16.5 million people with NIDDM and $14.9 billion for the 1 million people with IDDM [10]. 

 A recent global study indicated that the prevalence of diabetes mellitus was rising rapidly, particularly in developing countries [5]. Moreover, more than 60% of the population with diabetes mellitus all worldwide comes from Asia, as this remains the world's most populous region. The number of persons living with diabetes will increase substantially in each Asian country over the next few decades [5]. China is the world's largest developing economy and the most populous country, with one-fifth of the global population. Due to the rapid westernization of diet and lifestyle, China has one of the largest diabetes mellitus populations around the world [11]. The total number of people with diabetes in China is projected to increase from 20.8 million in 2000 to 42.3 million in 2030 [5]. Diabetes and its complications also result in significant economic burden among individuals, families, and health care systems. According to WHO estimates, China will lose $558 billion of its national income to heart disease, stroke, and diabetes from 2006 to 2015 [12]. Therefore, there is an urgent need to reduce the future diabetes burden by providing adequate financial resources and structures of health care delivery in China, particularly within the context of continued rapid urbanization [13].

 Diabetes can affect many organ systems throughout the body (e.g., nervous system, renal system, and eyes) and can lead to serious complications over time [14, 15]. Recently, a meta-analysis confirmed that individuals with diabetes mellitus have an approximately 2-fold higher risk of large-vessel disease, such as coronary heart disease (CHD) and stroke [16], and nonvascular mortality [17]. Thus, control of the growing prevalence of diabetes mellitus has been widely promoted in order to reduce the risk of large-vessel disease [18]. The pharmacological treatment of diabetes mellitus [19] and lifestyle modification [20] have been shown to decrease the incidence of diabetes mellitus. However, it is very important to first determine the prevalence of diabetes mellitus in the general population, the levels of awareness, treatment, and control of diabetes mellitus. The key elements of effective control include an improvement in the awareness of diabetes mellitus among both health professionals and the general population. Some studies [21–23] have revealed that more than half of the individuals with diabetes mellitus were unaware of the condition. Furthermore, the percentage of patients with diabetes mellitus who were treated and controlled to target levels was substantially low. Moreover, knowing factors associated with the conditions should be useful for health services and public health action in terms of management and prevention. An improvement in the awareness, treatment, and control of diabetes is, therefore, essential for the management and reduction of its prevalence. 

 Recently, a systematic review on the prevalence of diabetes was published which included 22 cross-sectional studies [24]. However, there was no information provided about the trends in the prevalence of diabetes and the awareness, treatment, and control of the disease. Also, this review only included studies published between 2000 and 2010. To date, there has been no systematic review or meta-analysis on the level of awareness, treatment, and control of diabetes in mainland China. Thus, the purpose of the current paper was to perform a systematic review and meta-analysis of the published literature regarding the prevalence, awareness, treatment, and control of diabetes in mainland China. The objectives of our study were (i) to estimate the trends in prevalence, awareness, treatment, and control of diabetes from 1979 to 2012 in mainland China; and (ii) to estimate the gender, location, and age distributions of patients with diabetes. This systematic review and meta-analysis could provide an overview of the epidemiology of diabetes in the past thirty years in China on the level above the provincial.

## 2. Materials and Methods

### 2.1. Data Sources and Search Strategy

 PUBMED, EMBASE, Chinese Biomedical Database (CBM), CNKI database, Chinese WanFang Database, and Chongqing VIP database were searched from the date of establishment up to February 2013 using the search terms “chronic disease,” “metabolic syndrome,” “diabetes,” “diabetes  mellitus,” “prevalence,” “epidemiology,” “awareness,” “treatment,” “control,” “cross-sectional survey,” “longitudinal study” and “China”. We also examined the reference lists from the articles identified.

*Selection Criteria*. All included studies were required to meet the following selection criteria: (i) cross-sectional or longitudinal studies that provided the prevalence, awareness, treatment, and control of diabetes in mainland China; (ii) based on population samples rather than volunteers; and (iii) the study population being representative of the provincial or national population. Exclusion criteria were (1) reviews, editorials, letters, commentaries, or reports; (2) articles repeating data from other articles that were already included; (3) self-reporting data; and (4) studies based on special populations, that is, physical examination crowds, industry, or occupational groups, ethnicity, or age groups.

### 2.2. Data Extraction

Data were extracted independently by two investigators. Any disagreements were resolved by discussion. We contacted the authors of eligible studies to request further or missing information if needed for subsequent analysis. Data regarding the first author, year of publication, study location, survey date, age range, sampling method, location (urban/rural), diagnostic criteria, diagnosis method, total sample size, total case size, gender distribution, age distribution, and prevalence, awareness, treatment, and control of the diabetes were extracted wherever available. An awareness of DM was considered a self-reported previous diagnosis of DM by a physician or other healthcare practitioner among participants with DM. Treatment of DM was determined as self-reported if taking oral hypoglycemic medications, using insulin, or other nonpharmacological treatments for the management of high glucose levels. The management of fasting plasma glucose (FPG) at levels lower than 7.0 rnmol/L (126 mg/dL) in patients of DM in treatment was defined as control of DM.

### 2.3. Statistical Analysis

STATA software version 11.1 (Stata, College Station, TX, USA) and Review Manager (RevMan) version 5.1 [25] were used to calculate the pooled prevalence, awareness, treatment, and control of diabetes from all of the eligible studies. A summary of the prevalence, awareness, treatment, and control estimates was obtained using random-effects meta-analysis. Statistical heterogeneity was assessed using the *I* ^2^ statistic [26]. Subgroup analyses included gender, location, and age distribution of diabetes.

## 3. Results

### 3.1. Results of the Search

We identified 45,947 references through electronic searches of PUBMED (*n* = 3,541), EMBASE (*n* = 4,013), Chinese Biomedical database (CBM) (*n* = 9,547), China National Infrastructure database (CNKI) (*n* = 8,189), Chinese WanFang database (*n* = 9,165), and Chongqing VIP database (*n* = 11,492). After excluding duplicates and following examination of titles, abstracts, and full texts, 172 potentially eligible studies were identified. In addition, with a manual search of references, two more studies [27, 28] were obtained from the papers. Finally, there were 174 publications included for further analysis. Among these studies, 57 included repetitive data, 54 studies were researched in specific populations, and 7 studies were self-reports of diabetes prevalence. Therefore, we obtained 56 studies in total that were suitable for inclusion in our study [21–23, 27–79]. Among the included studies, one study [77] contained survey data for two periods, with the data for 2002-2003 being duplicated in another study [43]. All of the included trials were published from 1980 to 2012. 

### 3.2. Characteristics of the Included Studies

We summarized the characteristics of the 56 included studies which contained 7 nationwide studies and 49 provincial studies conducted in the general Chinese population. Among the included studies, there were 56 concerning the prevalence of diabetes, 10 regarding the awareness of diabetes, 8 involving the treatment of diabetes, and 8 regarding the control of diabetes. A total population of 2,058,243 individuals was investigated, and 120,992 patients with diabetes were detected. The 56 studies were conducted among all of the provinces of mainland China, and included 22 provinces, 4 municipalities, and 5 autonomous regions. Four studies were conducted among all ages, with the age of participants in 9 studies being greater than 30 years old; 3 studies did not provide information regarding the age range investigated. The sampling methods applied in the studies included random sampling, stratified sampling, cluster sampling, multistage-stratified sampling, or combinations of these and general surveys. In terms of diagnostic criteria for diabetes, 7 studies used the American Diabetes Association criteria (ADA) from 1997 or 2009, 31 studies used the criteria of the World Health Organization (WHO) from 1985, 1988, 1995, or 1999, one study used the International Diabetes Federation criteria (IDF) from 2005, and one study was performed according to the criteria outlined in the Lanzhou conference on diabetes in China; however, 12 studies did not provide clear information on the diagnostic criteria employed. Eighteen studies used fasting plasma glucose (FPG) and oral glucose tolerance tests (OGTT) as the methods of diagnosis, 17 studies used FPG alone, 13 studies used OGTT, and one study used FPG, OGTT, and urine glucose tests. The prevalence of diabetes in 56 studies varied from 0.61% to 20.85%. Levels of awareness, treatment, and control ranged from 28.50 to 62.54%, 17.72 to 92.50%, and 6.86 to 35.87%, respectively (Table 1).

### 3.3. Trends in the Prevalence of Diabetes

**Figure 1:**
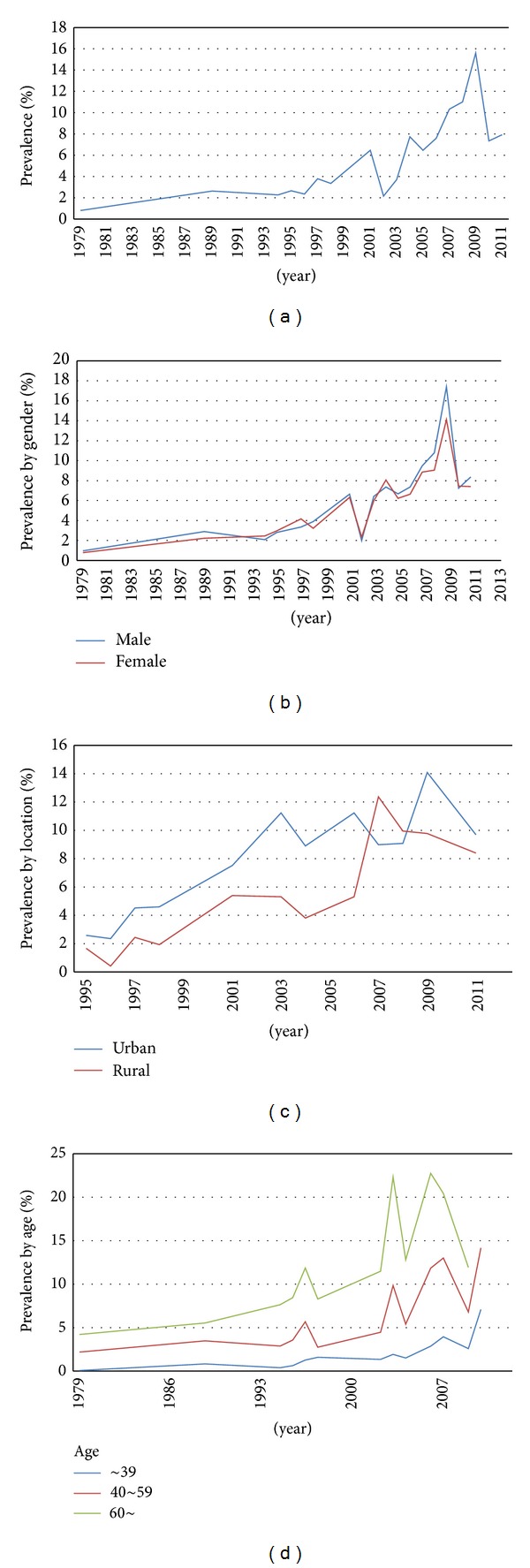
Trends in prevalence of diabetes and subgroup analysis by gender, location, and age.

As shown in Table 2, the overall prevalence of diabetes was 6.41% (95%CI: 5.50–7.33). The prevalence of diabetes by year varied from 0.81% to 15.60%. Table 2 and Figure 1(a) illustrate the trend in the overall prevalence of diabetes in mainland China from 1979 to 2012, showing that it increased as time progressed. The lowest prevalence of 0.81% was found in 1979 initially and showed a stable increase until 2001. After a slight decrease, the prevalence increased quickly from 2002 to 2009 (with the highest prevalence of 15.60% in 2009), before decreasing rapidly again from 2009 to 2012.

Forty-three studies reported the prevalence of diabetes by gender. The prevalence of diabetes in males was 6.91% (95%CI = 5.72–8.09), and the prevalence in females was 6.43% (95%CI = 5.12–7.74); there was no significant difference in the prevalence of diabetes between males and females (OR = 1.07, 95%CI = 0.98–1.16). Overall, trends in the prevalence of diabetes between males and females were increased and similar to the overall trend for the prevalence of diabetes; no significant differences could be observed between males and females (Figure 1(b)).

 Fifteen studies provided the prevalence of diabetes by location. The prevalence of diabetes in urban and rural areas was 7.82% (95%CI = 5.93–9.72) and 6.26% (95%CI = 4.25–8.27), respectively. A statistically significant difference could be found in the prevalence of diabetes between urban and rural areas (OR = 1.61, 95%CI = 1.25–2.06). As shown in Figure 1(c), trends in the prevalence of diabetes in both urban and rural areas increased over time, and the prevalence of diabetes in urban areas was consistently higher than that in rural areas.

 There were 26 articles which reported the prevalence of diabetes stratified by age. The prevalence of diabetes in the groups aged less than 39, 40–59, and over 60 was 1.98%, 6.96%, and 13.24%, respectively. Table 2 and Figure 1(d) show the information and trends in the prevalence of diabetes by age. An increasing tendency could be observed in all age groups over time. Also, the prevalence of diabetes increased with age (Figure 1(d)). 

### 3.4. Trends in the Awareness of  Diabetes

**Figure 2:**
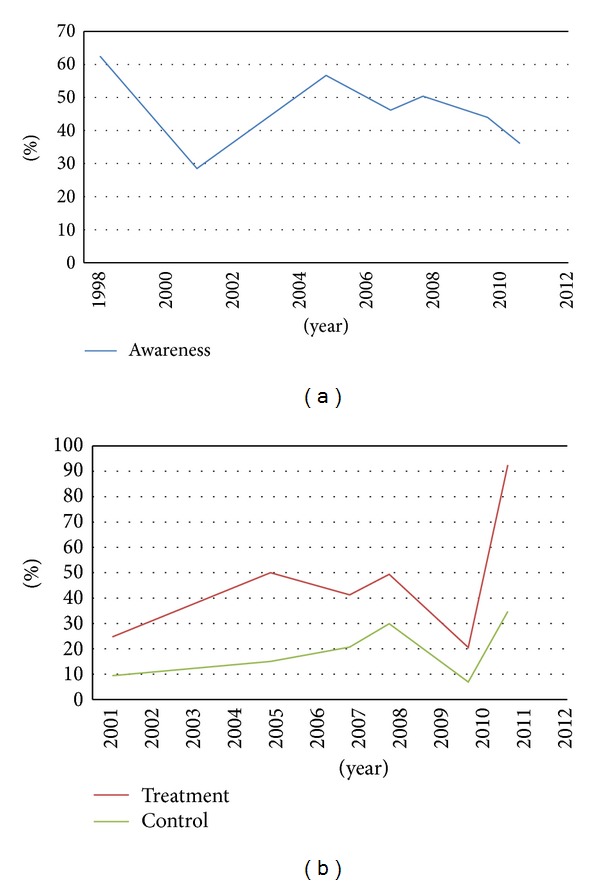
Trends in awareness, treatment, and control of diabetes.

 Ten studies provided information on awareness of diabetes. The pooled estimate for the awareness of diabetes was 45.81% (95%CI = 37.88–53.74) (Table 3). However, no obvious increasing trend could be observed in awareness of diabetes from 1998 to 2012 (Figure 2(a)).

Seven studies reported the awareness of diabetes by gender. Overall, the rate of awareness of diabetes in males was 40.86% and was 41.58% for females. No significant difference could be found in the awareness of diabetes between males and females (OR = 0.99, 95%CI = 0.87–1.13) (Table 3).

 Four studies reported the awareness rate of diabetes for urban and rural populations. The awareness rate in urban areas was 44.25% (95%CI = 32.6–55.9), while it was 34.27% (95%CI = 21–47.54) in rural locations (Table 3).

### 3.5. Trend in the Treatment of Diabetes

The combined result for the treatment rate of diabetes was 42.54% (95%CI = 13.69–71.38) (Table 3). As shown in Figure 2(b), an increasing tendency in the treatment of diabetes could be observed from 2001 to 2005. However, it decreased quickly after 2008 and then rapidly increased again from 2010 to 2011.

 Six studies provided the treatment rate of diabetes stratified by gender. The treatment rate of diabetes in males was 38.48%, whereas it was 41.18% for females. Comparing the treatment rate of diabetes for males to that of females, no significant difference could be observed (OR = 0.9, 95%CI = 0.72–1.14) (Table 3).

 Three studies reported the treatment rate by location. The treatment rates in urban and rural areas were 50.18% (95%CI = 31.22–100.39%) and 45.42% (95%CI = −12.7% and −103.6%), respectively (Table 3). Comparing the treatment rate of diabetes between rural and urban areas, there was no significant difference (OR = 1.30, 95%CI = 0.77–2.20).

### 3.6. Trends in the Control of Diabetes

 The pooled control rate was 20.87% (95%CI = 10.76–30.97) for diabetes from the combined information of eight studies (Table 3). There was an increasing tendency in the control of diabetes from 2001 to 2008, whereas the trend of control from 2008 to 2011 was similar to that of the treatment (Figure 2(b)).

 Five studies reported the control of diabetes stratified by gender. Overall, the control rate of diabetes in males was 19.26% and was 19.03% for females. There was no significant difference between males and females (OR = 1.06, 95%CI = 0.98–1.15) (Table 3).

 The control rate of diabetes in urban areas was 16.7% (95%CI = −2.39–35.8%) and 18.59% (95%CI = −1.2–38.39) in rural locations. No significant differences were found between urban and rural populations (OR = 18.59, 95%CI = −1.20–38.39) (Table 3). 

## 4. Discussion

Due to the rapid development of the economy in China, the dietary habits and lifestyles of individuals have changed remarkably, which might have contributed to the increase in many chronic diseases such as diabetes [11, 80]. Overall, the results of our study showed that the prevalence, awareness, treatment, and control of diabetes in mainland China were 6.41%, 45.81%, 42.54%, and 20.87%, respectively. A higher prevalence of diabetes was found in urban areas than in rural locations. As for the awareness of diabetes, this was also higher among residents in urban than in rural areas. There were no significant differences when data were stratified by gender or location in the subgroup analysis with regard to the treatment and control of diabetes. To the best of our knowledge, our study is the first systematic review on the awareness, treatment, and control of the diabetes and covers the longest time period from 1979 to 2012 in mainland China on the level of survey above the provincial.

The diabetes prevalence in our study ranged from 0.61% [27] to 20.85% [69] with an average of 6.41%; however, many authors have shown different findings when studying other countries in Asia. Similar to our results, it was reported that the prevalence of diabetes in Indonesia is 5.7%. In contrast, a survey conducted in Vietnam indicated a prevalence of 1.4% to 2.5%, which is relatively lower than that of our study [81]. On the contrary, epidemiological data from Thailand and Japan show that diabetes prevalence estimates were approximately 11.9% and 10%, which were higher than our study [82, 83]. The wide variation in the prevalence could be explained by the heterogeneity of the studies, geographical differences, and the application of different diagnostic and sampling methods. According to the statistics of the IDF, diabetes affects about 100 million people worldwide. By 2007, the number of affected individuals had reached 246 million [84]. From 1979 to 2002, nationwide epidemiological surveys of diabetes were conducted in China and the prevalence showed a 5-fold increase [27, 32, 85–87]. In accordance with this, an increasing trend in the prevalence of diabetes was found in our study. However, limited studies were included for pooled prevalence estimates; therefore, more studies with high quality and larger sample sizes are required to further confirm our findings.

 As to the prevalence by gender, no significant difference could be found in our study between males and females, which is similar to the results of most studies according to the International Diabetes Federation (IDF) [84]. However, the study reported by Yang et al. in 2010 [72] showed a difference in the prevalence of diabetes between males and females. This might partly be explained by the fact that men are more accustomed to unhealthy diets due to their work and also smoke more frequently than women, which are the main risk factors for diabetes. Moreover, men are much more careless with regard to their health. However, further studies are required to confirm our findings. 

 In the analysis stratified by location, the prevalence of diabetes in urban areas was higher than in rural locations, with an odds ratio of 1.61 for residents in urban and rural areas, which suggests that residents in urban areas were more likely to suffer from diabetes than those in rural regions. In accordance with our findings, many studies worldwide have also reported a higher prevalence of diabetes in urban areas than in rural populations [32, 86, 88]. With the rapid development of the economy, urbanization has become a current trend in developing countries [89]. Urbanization is related to the change in the food supply and lifestyles which could lead to an unhealthy diet, sedentary habits, and overnutrition [72, 90]. The prevalence of overweight and obesity has increased over the past two decades, especially in urban areas and high-income groups, which are the risk factors for diet-related disease and contribute to the development of diabetes [80].

 Age is an important risk factor for the development of diabetes [91–93]. It was reported that the prevalence of diabetes increased with age [85, 86]. A nationwide epidemiology survey showed that prevalence of diabetes in the age group of 20–39 was 1.01%, while it reached 6.31% in the age group over 40; however, the highest prevalence of 11.34% could be found in the age group over 60 [85]. Results of our study further confirm these findings. With the largest population in the world, China has become an ageing country; therefore, the prevalence of diabetes will increase as a result of the aging population in China. The health care for the aged population needs more attention from the government.

 Ten studies provided information on the awareness of diabetes. Among the included study subjects, 45.81% were aware of having diabetes. From 1998 to 2011, there was no obvious improvement in the awareness rate of diabetes. However, we report a difference between the urban and rural areas when conducting stratified analysis by location. This difference in awareness may be a result of a lack of access to health care and knowledge in rural areas. Increasing the awareness through health education remains an effective method for the prevention and treatment of diabetes. 

 Among the participants who had diabetes in our study, 42.54% of them were undergoing treatment. This is high compared with the study which first reported the treatment rate of diabetes (27.2%) in China [94]. The results of our study also showed an increasing trend in the treatment rate of diabetes from 2001 to 2011. However, no gender or location disparity was observed. The treatment of diabetes is not merely related to public awareness of the disease. More importantly, diagnostic methods play an essential role in the treatment of diabetes. Convenient, economic, and effective detection tools could improve the treatment rate of this disease.

 It was reported that controlling blood glucose at a normal level can prevent many diabetes-related complications, such as retinopathy, nephropathy, neuropathy, and macroangiopathy [95]. Our results indicated an increasing trend in the control of diabetes. Also, no gender or location differences were found in the subgroup analysis. The control rate of diabetes in our study is 20.87%, which is higher than that reported from a previous study with a control rate of 9.7% [94], but lower than that identified in another study (40.3%) [96]. The control of diabetes can be influenced by many factors, for example, obesity, smoking, insufficient exercise, and genetic susceptibility. Therefore, diversity exists in the control rate of diabetes in different places. Regular, effective, and timely treatment is primary in diabetes control. Moreover, adjusting dietary habits, changing lifestyles, and giving up smoking are essential when taking the risk factors for diabetes into account. 

 Coory [97] stated that it was very difficult to avoid heterogeneity in a meta-analysis, and this is also true for the meta-analysis of data from epidemiologic studies due to methodological problems. There were several methodological problems that might help to explain the heterogeneity: (1) different degrees of urbanization and socioeconomic conditions existed; (2) the included studies were carried out in different cities at various time points; (3) large differences existed in the age range, sampling methods, sample sizes, and response rates; (4) different diagnostic criteria and diagnosis methods were used in studies, with other studies not mentioning them at all; and (5) not all of the included studies provided sufficient information on gender, location, and age for subgroup analysis.

 There are some limitations of the current study which need to be highlighted. Firstly, the present study might have underestimated the true prevalence of diabetes, since some of the studies included in the present review only used fasting plasma glucose (FPG) levels to diagnose new cases of diabetes; also, some studies did not provide any information about the diagnostic methods. Thus, more accurate measurements in the detection of undiagnosed diabetes are necessary in future research, for example, the two-hour glucose tolerance test and the oral glucose tolerance test (OGTT). Secondly, in addition to the subgroups of gender, location, and age, we did not report any positive risk factors associated with the prevalence of diabetes and the awareness, treatment, and control of the diabetes (e.g., overweight, obesity, hypertension, and hyperlipidemia); thus, we were unable to assess the association between them. Thirdly, the age range is quite different among the studies included. The age distribution in the sample population could markedly affect the results, since some locations contain a large number of individuals >40 years old; therefore, the prevalence of diabetes in some studies is likely to be higher than in others. Fourthly, limited studies were included in our meta-analysis for the pooled estimates; for example, only one study was included for analysis in 2009 and 2012 as studies from that time are possibly yet to be published. This could also be seen with regard to the trends in awareness of diabetes in 2001 and in the treatment and control of diabetes in 2011. Therefore, more studies were required for further analysis. A further limitation of the present study is that information in the subgroup analysis of awareness, control, and treatment by gender, location, and age was limited; therefore, we did not perform the relevant trend analyses.

The study showed an increasing trend for the prevalence, treatment, and control of diabetes among the population of mainland China from 1979 to 2012, but not in the awareness. This review will help us understand the gaps in the current research, which is useful for investigators and health care providers regarding aboriginal health. Thus, urgent measures are needed to prevent the high prevalence of diabetes and to improve diabetes awareness, control, and treatment among the Chinese populations.

## Figures and Tables

**Table 1:** Characteristics of the included studies.

First author and survey year	Study location	Survey date	Survey level	Age range	Sampling method	Location	Diagnostic criteria	Diagnosis method	Total sample size (male/female)	Total case size (male/female)	Prevalence (%)	Awareness (%)	Treatment (%)	Control (%)
Sha 1980 [29]	Shanghai	1978~1979	Provincial	0~89	NA	Urban/rural	NA	Urine glucose/FPG/ OGTT	101624 (40664/51960)	1028 (523/505)	1.01 (0.95~1.07)	*∖*	*∖*	*∖*
Zhong 1979 [34]	China	Oct. 1979	National	All age	Cluster sampling	Urban/rural	The Lanzhou conference on diabetes	OGTT	304537 (160195/144342)	1854 (1009/845)	0.61 (0.58~0.64)	*∖*	*∖*	*∖*
Xiang 1989 [36]	Shanxi/ Beijing/ Liaoning	1989	Provincial	20~	General survey	Urban/rural	WHO1988	OGTT	44747 (27938/16809)	1181 (808/373)	2.64 (2.49~2.79)	*∖*	*∖*	*∖*
Pan 1994 [32]	China	Apr. 1994	National	25~64	General survey	Urban/rural	WHO1985	OGTT	213515	4864	2.28 (2.21~2.34)	*∖*	*∖*	*∖*
Xu 1994 [31]	Yunnan	1994	Provincial	15~	Stratified random cluster sampling	Urban/rural	NA	OGTT	4252 (1963/2289)	97 (41/56)	2.28 (1.83~2.73)	*∖*	*∖*	*∖*
Liu 1995 [35]	Ningxia	1995	Provincial	35~	Stratified random cluster sampling	Urban	BG ≥ 7.2 mmol/L/history of diabetes	FPG	2039 (1092/947)	77 (34/43)	3.78 (2.95~4.60)	*∖*	*∖*	*∖*
Wang 1995 [39]	Hubei	July. 1994~Mar. 1995	Provincial	25~70	Stratified random cluster sampling	Urban/rural	WHO1985	OGTT	9450 (4790/4660)	248 (137/111)	2.62 (2.30~2.95)	*∖*	*∖*	*∖*
Yang 1995 [33]	Anhui	Sept. 1994~Aug. 1995	Provincial	15~80	Stratified cluster sampling	Urban/rural	WHO1985	OGTT	10991 (6132/4859)	246 (150/96)	2.24 (1.96~2.51)	*∖*	*∖*	*∖*
Zhang 1995 [34]	Fujian	1995	Provincial	15~	NA	Urban/rural	BG ≥ 11.1 mmol/L	OGTT	4075	83	2.04 (1.60~2.47)	*∖*	*∖*	*∖*
Xiang 1996 [36]	China	1995~1996	National	20~75	Stratified cluster sampling	Urban/rural	WHO1985	FPG	42751	1548	3.62 (3.44~3.80)	*∖*	*∖*	*∖*
Zhao 1996 [38]	Shandong	1994~1996	Provincial	0~99	Stratified random cluster sampling	Urban/rural/other	WHO	OGTT	20228	221	1.09 (0.95~1.24)	*∖*	*∖*	*∖*
Liu 1997 [42]	Hebei	Feb.~June. 1997	Provincial	20~74	Stratified random sampling	Urban/rural	WHO1985	FPG	5975 (2771/3204)	287 (117/170)	4.80 (4.26~5.35)	*∖*	*∖*	*∖*
Zhang 1997 [37]	Hebei	Nov. 1996~Feb. 1997	Provincial	20~74	Stratified random cluster sampling	Urban/rural	NA	FPG/OGTT	2762 (1207/1555)	77 (30/47)	2.79 (2.17~3.40)	*∖*	*∖*	*∖*
Sheng 1998 [40]	Shanghai	1998	Provincial	30~79	General survey	Urban	ADA1997/ WHO1985	FPG/OGTT	9376 (4096/4307)	299 (168/131)	3.19 (2.83~3.54)	62.54 (57.06~68.03)	*∖*	*∖*
Wang 1998 [41]	Guangdong	1997~1998	Provincial	20~74	Stratified random cluster sampling	Urban/rural	WHO1985	OGTT	11742 (5450/6292)	414 (200/214)	3.53 (3.19~3.86)	*∖*	*∖*	*∖*
Hu 2001 [21]	China	2000~2001	National	35~74	A four-stage stratified sampling random cluster method	Urban/rural	ADA1997	FPG	15236 (7368/7868)	986 (489/497)	6.47 (6.08~6.86)	28.50 (25.68~31.32)	24.75 (22.05~27.44)	9.43 (7.61~11.26)
Gu 2002 [43]	Shanghai	Aug. 2001~Jan. 2002	Provincial	15~	Multistage-stratified cluster sampling	Urban	NA	NA	7563 (3691/3872)	131 (53/78)	1.73 (1.44~2.03)	*∖*	*∖*	*∖*
Wang 2002 [46]	China	Aug.~Dec. 2002	National	18~	Multistage-stratified random cluster sampling	Urban/rural	WHO1995	FPG/OGTT	52416 (24428/27988)	1364 (620/744)	2.60 (2.47~2.74)	*∖*	*∖*	*∖*
Hu 2003 [47]	Zhejiang	2003	Provincial	All age	Multistage-stratified random cluster sampling	NA	NA	NA	21666 (10896/10770)	141	0.65 (0.54~0.76)	*∖*	*∖*	*∖*
Li 2003a [45]	Sichuan	2003	Provincial	All age	Stratified cluster sampling	Urban/rural	NA	NA	12714 (6403/6311)	320	2.52 (2.24~2.79)	*∖*	*∖*	*∖*
Li 2003b [49]	Shanghai	2003	Provincial	15~	A four-stage stratified random cluster sampling	Urban/rural	WHO1999	OGTT	11589 (4621/6968)	1000 (412/588)	8.63 (8.12~9.14)	*∖*	*∖*	*∖*
Ma 2003 [44]	Qinghai	2001~2003	Provincial	20~74	Stratified cluster sampling	Urban/rural	ADA/ WHO1997	FPG/OGTT	2276	70	3.08 (2.37~3.78)	*∖*	*∖*	*∖*
Zhang 2003 [62]	Inner Mongolia	July. 2002~Sept. 2003	Provincial	20~	NA	Rural	ADA 2009	FPG	2563 (1050/1513)	94 (41/53)	3.67 (2.94~4.40)	*∖*	*∖*	*∖*
Sun 2004 [48]	Hebei	Sept.~Oct. 2004	Provincial	18~69	Multistage stratified sampling	Urban/rural	FPG ≥ 7.0 mmol/L/ 2 hBG ≥ 11.1 mmol/L/ previous diagnosis of diabetes	FPG/OGTT	4196 (1731/2465)	224 (94/130)	5.34 (4.66~6.02)	*∖*	*∖*	*∖*
Tian 2004 [59]	Tianjin	2004	Provincial	35~	NA	Rural	WHO1999	FPG	769792 (364781/405011)	72803 (30277/42526)	9.50 (9.43~9.57)	*∖*	*∖*	*∖*
Zhao 2004 [52]	Shandong	2004	Provincial	20~74	Stratified random cluster sampling	Urban/rural	WHO1999	FPG/OGTT	5003 (2395/2608)	419 (200/219)	8.38 (7.61~9.14)	*∖*	*∖*	*∖*
Qi 2005 [74]	Tianjin	June. 2005	Provincial	15~	Multiphase stratified random cluster sampling	NA	ADA1997/ Immunology of Diabetes Society criteria	FPG	8109 (3878/4231)	498 (299/199)	6.14 (5.62~6.66)	*∖*	*∖*	*∖*
Wei 2005 [71]	Heilongjiang	2005	Provincial	20~	Multistage random cluster sampling	Rural	WHO1999	FPG/OGTT	1058 (530/528)	75 (38/37)	7.09 (5.54~8.64)	*∖*	*∖*	*∖*
Zhang 2005 [51]	Beijing	Sept.~Nov. 2005	Provincial	18~	Stratified multi-stage cluster sampling	Urban/rural	WHO1999	FPG	16658 (6605/10053)	1099	6.60 (6.22~6.97)	56.69 (53.76~59.62)	49.95 (47.00~52.91)	15.01 (12.90~17.13)
Zhi 2005 [57]	Tianjin	June.~Sept. 2005	Provincial	15~74	Multiphase stratified cluster sampling	Urban/rural	WHO1999/IDF	FPG	20741 (9986/10755)	1259 (511/748)	6.07 (5.75~6.40)	*∖*	*∖*	*∖*
Li 2006 [50]	Shanghai	2006	Provincial	15~74	Multistage cluster random sampling	Urban/rural	WHO1999	OGTT	11589 (4621/6968)	1000 (412/588)	8.63 (8.12~9.14)	*∖*	*∖*	*∖*
Xu 2006 [54]	Beijing	2001~2006	Provincial	45~	NA	Urban/rural	BG ≥ 7.0 mmol/L/self-reported diabetes/a history of drug treatment for diabetes	Blood biochemical analyses	3251 (1838/1413)	381	11.72 (10.61~12.83)	*∖*	*∖*	*∖*
Zhang 2006 [61]	Qinghai	2006	Provincial	NA	NA	Urban/rural	WHO1999	FPG/OGTT	4864 (2905/1959)	233	4.79 (4.19~5.39)	*∖*	*∖*	*∖*
Zhao 2006 [63]	Shanxi	Aug. 2005~Aug. 2006	Provincial	15~	Random cluster sampling	Urban/rural	WHO1999	FPG/OGTT	12111 (5324/6787)	638 (309/329)	5.27 (4.87~5.67)	*∖*	*∖*	*∖*
Cheng 2007 [56]	Shanghai	Mar. 2007	Provincial	18~	Random sampling	Urban/rural	NA	NA	2320 (980/1340)	215 (91/124)	9.27 (8.09~10.45)	*∖*	*∖*	*∖*
Dai 2007 [65]	Jiangsu	2007	Provincial	18~	A four-stage stratified random cluster sampling	Urban/rural	WHO	FPG	11833 (5210/6623)	609 (271/338)	5.10 (4.70~5.50)	*∖*	*∖*	*∖*
Jin 2007 [66]	Beijing	Sept.~Nov. 2007	Provincial	20~	Random cluster sampling	Urban/rural	WHO1999	FPG/OGTT	3484 (1390/2094)	510 (238/272)	14.64 (13.46~15.81)	*∖*	*∖*	*∖*
Lv 2007 [22]	Jiangsu	2007	Provincial	15~69	Multistage random cluster sampling	Urban/rural	NA	FPG	4318 (2007/2311)	363 (153/210)	8.41 (7.58~9.23)	33.06 (28.22~37.90)	27.27 (22.69~31.85)	9.09 (6.13~12.05)
Wang 2007 [60]	Guangdong	2006~2007	Provincial	20~	A four-stage sampling method	Urban/rural	WHO1999	OGTT	6033 (1930/4103)	639 (229/410)	10.59 (9.82~11.37)	*∖*	*∖*	*∖*
Wei 2007 [70]	Beijing	June.~Sept. 2007	Provincial	20~	Stratified random cluster sampling	Urban/rural	WHO1999	FPG/OGTT	5465	1060	19.40 (18.35~20.44)	*∖*	*∖*	*∖*
Yu 2007 [55]	Shandong	2007	Provincial	25~	Multistage-stratified random sampling	Rural	WHO1999	FPG/OGTT	16341 (6992/9349)	697 (289/408)	4.27 (3.40~4.58)	*∖*	*∖*	*∖*
Zhan 2007 [79]	Beijing	May.~Aug. 2007	Provincial	20~	Multistage random cluster sampling	Urban/rural	WHO1999	FPG	10054 (3687/6367)	1105 (408/697)	10.99 (10.38~11.60)	59.28 (56.38~62.17)	55.20 (52.27~58.14)	32.22 (29.46~34.97)
Chen 2008a [64]	Shanghai	2007~2008	Provincial	40~	Multistage-stratified random sampling	Urban	NA	FPG	619 (230/389)	113	18.26 (15.21~21.30)	*∖*	*∖*	*∖*
Chen 2008b [67]	Heilongjiang	Sept. 2007~Mar. 2008	Provincial	20~74	Multistage stratified cluster sampling	Urban/rural	WHO1999	FPG/OGTT	3058 (1219/1839)	265 (135/130)	8.67 (7.67~9.66)	*∖*	*∖*	*∖*
Fan 2008 [53]	Zhejiang	NA	Provincial	35~	Multistage-stratified random cluster sampling	Urban/rural	NA	FPG	6902 (2620/4282)	401 (162/239)	5.81 (5.26~6.36)	50.37 (45.48~55.27)	49.38 (44.48~54.27)	24.19 (20.00~28.38)
Li 2008 [68]	Guangdong	Jan. 2007~Dec. 2008	Provincial	All age	Stratified cluster sampling	Urban/rural	NCEP-ATP III 2005 criteria	FPG	1206 (875/331)	52 (40/12)	4.31 (3.17~5.46)	*∖*	*∖*	*∖*
Lin 2008 [58]	Fujian	July. 2007~May. 2008	Provincial	20~74	Multistage-stratified sampling	Urban/rural	IDF 2005	FPG/OGTT	3208 (1250/1958)	305 (126/179)	9.51 (8.49~10.52)	*∖*	*∖*	*∖*
Su 2008 [69]	Beijing	Sept. 2008	Provincial	NA	Multistage-stratified and systematic random cluster sampling	Urban	NA	NA	1511 (504/1007)	315 (112/203)	20.85 (18.80~22.90)	*∖*	*∖*	35.87 (30.58~41.17)
Yang 2008 [72]	China	June. 2007~May. 2008	National	20~	Multistage stratified sampling	Urban/rural	WHO1999	OGTT	46239 (18419/27820)	4372 (1952/2420)	9.70 (9.43~9.97)	*∖*	*∖*	*∖*
Li 2009 [77]	Shanghai	2009	Provincial	35~74	NA	Urban/rural	ADA 2009	FPG/OGTT	7423 (3461/3962)	1158 (601/557)	15.60 (14.77~16.43)	*∖*	*∖*	*∖*
Le 2010 [73]	Yunnan	2008~2010	Provincial	18~	Four-stage stratified random sampling	Urban/rural	FBG was 7.0 mmol/L/using antidiabetic medications/previous diagnosis of diabetes	FPG	10007 (4628/5379)	657 (260/397)	6.57 (6.08~7.05)	29.38 (25.89~)	23.44 (20.20~26.68)	*∖*
Yang 2010 [23]	Xinjiang	2007~2010	Provincial	35~	Stratified sampling method	Urban/rural	ADA 2009	FPG	14122 (6539/7583)	948 (506/442)	6.71 (6.30~7.13)	43.35 (40.20~46.51)	17.72 (15.29~20.15)	6.86 (5.25~8.47)
Ye 2010 [75]	Zhejiang	July.~Nov. 2010	Provincial	18~	Multiphase stratified random cluster sampling	Urban/rural	WHO1999	FPG/OGTT	17437 (8169/9268)	1529 (683/846)	8.77 (8.35~9.19)	59.19 (56.73~61.65)	*∖*	*∖*
CDC 2011 [28]	31provinces	2011	National	18~	Multiphase stratified random cluster sampling	Urban/rural	NA	FPG/OGTT	96941 (44353/52588)	9403 (4524/4733)	9.70 (9.51~9.89)	36.10 (35.13~37.07)	92.50 (91.97~93.03)	34.70 (33.74~35.66)
Chen 2011 [76]	Shandong	July.~Sept. 2011	Provincial	18~69	Multiphase stratified random cluster sampling	Urban/rural	WHO1999	FPG/OGTT	15262 (7643/7619)	941 (499/442)	6.17 (5.78~6.55)	*∖*	*∖*	*∖*
Liu 2012 [78]	Ningxia	NA	Provincial	NA	Multistage random cluster sampling	NA	WHO1997	NA	3001 (1418/1583)	76	2.53 (1.97~3.09)	*∖*	*∖*	*∖*

NA: not available; FPG: fasting plasma glucose; OGTT: oral glucose tolerance test; BG: blood glucose concentration; WHO: World Health Organization; ADA: American Diabetes Association; IDF: International Diabetes Federation; NCEP-ATP: National Cholesterol Education Program Adult Treatment Panel.

**Table 2:** Trends in prevalence of diabetes in mainland China: 1979~2012.

Survey year	1979	1989	1994	1995	1996	1997	1998	2001	2002	2003	2004	2005	2006	2007	2008	2009	2010	2011	2012
Overall prevalence/*I* ^2^ (%)	6.41/99.8																		
Prevalence by year (%)	0.81	2.64	2.28	2.67	2.36	3.80	3.36	6.47	2.17	3.71	7.74	6.47	7.60	10.33	11.01	15.60	7.35	7.93	2.53
Gender (%)																			
Male	0.96	2.89	2.09	2.81	*∖*	3.35	3.89	6.64	1.99	6.41	7.36	6.67	7.36	9.48	10.79	17.39	7.24	8.36	*∖*
Female	0.78	2.22	2.45	2.97	*∖*	4.16	3.22	6.32	2.34	5.97	8.06	6.22	6.64	8.85	9.05	14.11	7.45	7.40	*∖*
Location (%)																			
Urban	*∖*	*∖*	*∖*	2.58	2.35	4.52	4.60	7.52	*∖*	11.23	8.90	*∖*	11.23	8.99	9.08	14.09	*∖*	9.69	*∖*
Rural	*∖*	*∖*	*∖*	1.66	0.42	2.44	1.93	5.40	*∖*	5.31	3.81	*∖*	5.31	12.37	9.95	9.78	*∖*	8.40	*∖*
Age (%)																			
~39	0.06	0.83	*∖*	0.39	0.63	1.27	1.59	*∖*	*∖*	1.35	1.92	1.51		2.87	3.94		2.59	7.09	
40~59	2.19	3.48	*∖*	2.88	3.57	5.68	2.74	*∖*	*∖*	4.46	9.83	5.39	*∖*	11.84	13.01	*∖*	6.79	14.16	*∖*
60~	4.21	5.53	*∖*	7.63	8.45	11.86	8.29	*∖*	*∖*	11.48	22.32	12.81	*∖*	22.75	20.44	*∖*	11.93		*∖*

**Table 3:** Overall awareness, treatment, and control of diabetes and subgroup analysis.

Awareness						
Overall awareness	The number of studies	Case	Population	Awareness rate (%)	95% CI	*I* ^2^ (%)
	10	6,971	16,790	45.81	37.88~53.74	98.8
Subgroup analysis						
Gender						
Male	7	2,802	7,023	40.86	32.86~48.85	97.0
Female	7	3,126	7,822	41.58	31.47~51.70	98.5
Location						
Urban	4	2,429	5,231	44.25	32.60~55.90	98.0
Rural	4	2,037	6,284	34.27	21.00~47.54	98.5

Treatment						
Overall treatment	The number of studies	Case	Population	Treatment rate (%)	95% CI	*I* ^2^ (%)
	8	10,720	14,962	42.54	13.69~71.38	99.9

Subgroup analysis						
Gender						
Male	6	4,715	6,340	38.48	0.64~76.32	99.9
Female	6	5,117	6,976	41.18	6.96~75.40	99.9
Location						
Urban	3	3,649	4,448	50.18	31.22~100.39	99.8
Rural	3	4,680	5,538	45.42	−12.7~103.60	99.9

Control						
Overall control	The number of studies	Case	Population	Control rate (%)	95% CI	*I* ^2^ (%)
	8	922	14,620	20.87	10.76~30.97	99.5

Subgroup analysis						
Gender						
Male	5	1,737	5,784	19.26	4.08~34.45	99.4
Female	5	1,777	6,085	19.03	4.73~33.33	99.4
Location						
Urban	3	1,320	4,448	16.70	−2.39~35.80	99.5
Rural	3	1,809	5,538	18.59	−1.20~38.39	99.4
